# Functional Performance After Complex Endovascular Aortic Repair: A Single-Center Retrospective Cohort Study

**DOI:** 10.1177/15266028211028222

**Published:** 2021-06-30

**Authors:** Britt W. C. M. Warmerdam, Yara van Holstein, Daniël Eefting, Carla S. P. van Rijswijk, Rutger W. van der Meer, Simon P. Mooijaart, Jaap F. Hamming, Joost R. van der Vorst, Jan van Schaik

**Affiliations:** 1 Department of Surgery, Leiden University Medical Center, Leiden, The Netherlands; 2 Department of Gerontology and Geriatrics, Leiden University Medical Center, Leiden, The Netherlands; 3 Department of Radiology, Leiden University Medical Center, Leiden, The Netherlands

**Keywords:** complex aortic aneurysm repair, functional performance, activities of daily living, frailty, endovascular treatment/therapy

## Abstract

**Purpose:**

Complex endovascular aortic repair (EVAR) procedures provide a treatment option for patients with aortic aneurysms involving visceral branches. Good technical results and short-term outcomes have been reported. Whether complex EVAR provides acceptable *functional* outcomes is not clear. The current study aims to describe postoperative functional outcomes in complex EVAR patients—an older and relatively frail patient group.

**Materials and Methods:**

A single-center retrospective cohort study was performed, using data from a computerized database of consecutive patients who underwent complex EVAR in the Leiden University Medical Center (LUMC, The Netherlands) between July 2013 and September 2020. As of May 2017, patients scheduled for complex EVAR were referred to a geriatric care pathway to determine (Instrumental) Activities of Daily Living ((I)ADL) scores at baseline and, if informed consent was given, after 12 months. For the total patient group, adverse functional performance outcomes were: discharge to a nursing home and 12-month mortality. For the patients included in geriatric follow-up, the additional outcome was the incidence of functional decline (defined by a ≥2 point increase in (I)ADL-score) at 12-month follow-up

**Results:**

Eighty-two patients underwent complex EVAR, of which 68 (82.9%) were male. Mean age was 73.3 years (SD=6.3). Within 30 days postsurgery, 6 patients (7.3%) died. Mortality within 12 months for the total patient group was 14.6% (n=12). After surgery, no patients had to be discharged to a nursing home. Fifteen patients (18.3%) were discharged to a rehabilitation center. Twenty-three patients gave informed consent and were included in geriatric follow-up. Five patients (21.7%) presented functional decline 12 months postsurgery and 4 patients had died (17.4%) by that time. This means that 39.1% of the patients in the care pathway suffered an adverse outcome.

**Conclusion:**

To our knowledge, this is the only study that examined functional performance after complex EVAR, using a prospectively maintained database. No patients were newly discharged to a nursing home and functional performance results at 12 months are promising. Future multidisciplinary research should focus on determining which patients are most prone to deterioration of function, so that efforts can be directed toward preventing postoperative functional decline.

## Introduction

Endovascular aortic repair (EVAR) is well established in clinical practice for treating abdominal aortic aneurysms (AAA) located below the visceral arteries.^1,2^ Because of low immediate morbidity and mortality rates compared with open surgical repair (OSR), EVAR is often the procedure of choice.^3,4^ In recent years, endovascular techniques for aortic repair have developed extensively. Fenestrated EVAR (FEVAR) and branched EVAR (BEVAR) allow for endovascular treatment of complex aortic aneurysms, comprising segments of the entire aortoiliac tract, including the arch.^5^

These techniques have greatly expanded treatment options. Patients with complex aortic aneurysms who are considered too frail for OSR because of (cardiopulmonary) comorbidities, decreased physical performance or other factors increasing the risk of adverse outcomes, can now also be operated using a less invasive endovascular approach. These extensive EVAR procedures have a higher morbidity and mortality risk than conventional infrarenal EVAR. Thirty-day mortality rates ranging from 3.4% up to 8.6% have been reported in complex EVAR, compared with an average of 1.2% in conventional EVAR.^6–9^

Besides morbidity and mortality, patients undergoing complex EVAR are at risk of decline in functional performance. Living independently of care and maintaining quality of life are highly valued outcomes, especially in older patients.^10–12^ While high technical success rates are commonly reported, evidence on functional performance after complex EVAR is scarce.^13,14^ Information on postoperative functional performance is important in order to properly inform patients about the consequences of treatment. We evaluated the functional outcome of patients undergoing complex EVAR in a tertiary referral center. To our knowledge, this study is the only study that examined functional performance after complex EVAR, using a prospectively maintained database.

## Materials and Methods

### Study Design and Setting

A single-center retrospective cohort study was performed, using data from a prospectively maintained secure computerized database of consecutive patients who underwent complex EVAR in the Leiden University Medical Center, a tertiary referral center (LUMC, The Netherlands). Patients were included since the introduction of complex EVAR in this hospital in July 2013, until September 2020. The database was approved by the LUMC Medical Ethics Committee (METC). Any information not provided by this database was subtracted from patients’ medical records. As in the standard care pathway, all patients were seen at 6 weeks, 6 months, and 12 months postsurgery and yearly after that for outpatient-based follow-up. Additional appointments were made if deemed necessary. Computed tomography angiography, duplex ultrasonography, and abdominal X-ray were used in follow-up. Given the retrospective character of the current study, the METC waived the necessity for informed consent.

As of May 2017, all patients scheduled for complex EVAR were referred to the LUMC geriatric department to undergo a comprehensive geriatric assessment. No selection based on patient demographics was made. This included (Instrumental) Activities of Daily Living Scores ((I)ADL), the 6-item Cognitive Impairment Test (6-CIT), and a Mini Nutritional Assessment (MNA). If the patient gave informed consent for follow-up, they were included in the Triage of Elderly Needing Treatment (TENT) study (ID number: NL53575.058.15).^15^ For these patients, geriatric scores were gathered again at 12 months postsurgery, by phone. In the current study, (I)ADL-scores were used to examine functional performance at 12 months.

### Patients and Procedures

Complex EVAR was defined as endovascular aortic surgery that entailed correction of an aneurysm including the visceral segment, with or without thoracic involvement. Baseline characteristics were described by demographics, living status, aneurysm characteristics, comorbidities, risk factors, and exercise tolerance by the estimated metabolic equivalent of task (MET) score.^16^ (I)ADL-scores were measured by the Katz Index of Independence in Activities of Daily Living (Katz ADL) and the Lawton Instrumental Activities of Daily Living Scale (Lawton IADL).^17,18^ The Katz ADL measures the (in)dependency of patients with regard to 6 daily life activities: bathing, getting dressed, toileting, transfers, continence, and feeding. For each activity, patients can score 0 (fully independent) to 2 (dependent). Patients are categorized on a hierarchic 0 to 12 scale, with 0 being independent and 12 being fully dependent in all 6 activities. The Lawton IADL measures the (in)dependency of patients with regard to 8 more complex activities: using the phone, shopping, preparing food, housekeeping, doing laundry, mode of transportation, responsibility for personal medication, and handling finances. Patients are categorized on a 0 to 24 scale, scoring 0 (fully independent) to 3 (not capable/has never performed) per category. The 6-CIT and MNA were used to examine cognitive impairment (score >7) and malnutrition risk (score <11), respectively.^19–21^ Both the Katz ADL and Lawton IADL are of sufficient validity when conducted by phone.^22,23^

### Outcomes

Adverse outcome measures for the total patient group were postoperative discharge to a nursing home and mortality at 12 months postsurgery. Clinical outcomes were: 30-day mortality, length of hospital stay, and major surgical complications defined as complications with a Clavien-Dindo score of III-IV.^24^

For the 23 complex EVAR patients included in the TENT-study, the additional adverse outcome was the incidence of functional decline at 12 months postsurgery. This was defined as an increase of at least 2 points on the Katz ADL and/or Lawton IADL scale. This entails the patient becoming (more) dependent in at least one category and is in line with definitions of functional decline used in other fields.^25–27^

### Statistical Methods

Baseline characteristics were expressed by number of patients and percentages, or as mean with the standard deviation (SD) in case of normal distribution. In case of skewed distribution, characteristics were presented as median with the interquartile range (Q1, Q3). Patients who did give informed consent for geriatric follow-up were compared with patients who did not consent, based on baseline characteristics. The independent *t* test was used for continuous normally distributed data, chi-square test for categorical data, and the Mann-Whitney *U* test for skewed data. All analyses were made using IBM SPSS Statistics version 26.

## Results

### Patient Characteristics

**Figure 1.**
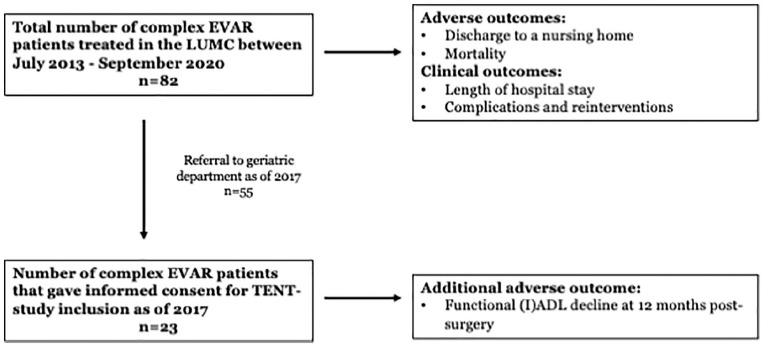
Patient inclusion and study outcomes. EVAR, endovascular aortic repair; (I)ADL, (Instrumental) Activities of Daily Living; LUMC, Leiden University Medical Center; TENT, Triage of Elderly Needing Treatment.

A total of 82 consecutive patients who underwent complex EVAR were included. Figure 1 shows a flowchart of patient inclusion. Table 1 shows the baseline characteristics of these patients; 68 (82.9%) were male, with a mean age of 73.3 years (SD=6.3). Mean aneurysm size was 65.1 mm (SD=11.1) and 17 patients (20.7%) had undergone previous aortic repair surgery (open or endovascular). Most patients (n=77, 93.9%) were hospitalized from home. Three patients (3.7%) were living in a nursing home, 1 patient (1.2%) lived in a homeless shelter and 1 patient (1.2%) had an unknown living situation at admission. The median ADL baseline score was 0.0 (IQR=0.0, 0.0) and the median IADL score was 1.0 (IQR=0.0, 3.75). Aneurysms were treated using FEVAR (59.8%), BEVAR (25.6%), FBEVAR (11.0%, using a graft with fenestrations and branches) and Arch-FEVAR (3.7%).

**Table 1.** Patient Characteristics at Baseline.

Variable (Unit)	Total Patient Group (n=82)
Age (years), mean (SD)	73.3 (6.3)
Male, n (%)	68 (82.9)
BMI (kg/m^2^), mean (SD)	26.7 (3.6)
Aneurysm size (mm), mean (SD)	65.1 (11.1)
Procedure type, n (%)	
FEVAR	49 (59.8)
BEVAR	21 (25.6)
FBEVAR	9 (11.0)
Arch-FEVAR	3 (3.7)
ASA score ≥3, n (%)	48 (58.5)
Comorbidities, n (%)	
Cardiac	53 (64.6)
Pulmonary	27 (32.9)
eGFR <60 mL/min/1.73m^2^, n (%)	33 (40.2)
CVA/TIA	20 (24.4)
Peripheral vascular disease	16 (19.5)
Diabetes mellitus type 2	11 (13.4)
Malignancy	
Active	3 (3.7)
Cured	20 (24.4)
Other comorbidities	26 (31.7)
Risk factors, n (%)	
Currently smoking	25 (30.5)
Hypercholesterolemia	26 (31.7)
Hypertension	59 (72.0)
Previous aortic repair, n (%)	17 (20.7)
Low tolerance of exercise (MET 1–4), n (%)	12 (14.6)
Living status, n (%)	
Home	77 (93.9)
Nursing home	3 (3.7)
Other/unknown	2 (2.4)
Baseline (I)ADL scores	n=40
ADL, median (IQR)	0.0 (0.0, 0.0)
IADL, median (IQR)	1.0 (0.0, 3.75)

Abbreviations: AAA, abdominal aortic aneurysm; ACS, acute coronary syndrome; ADL, activities of daily living; ASA, American Society of Anesthesiologists classification; BEVAR, branched endovascular aortic repair; BMI, body mass index; COPD, chronic obstructive pulmonary disease; CVA, cerebrovascular accident; eGFR, estimated glomerular filtration rate; FEVAR, fenestrated endovascular aortic repair; IADL, instrumental activities of daily living; IQR, interquartile range (Q1, Q3); MET, metabolic equivalent of task; MI, myocardial infarction; TIA, transient ischemic attack.

### Care Dependency at Discharge

**Figure 2.**
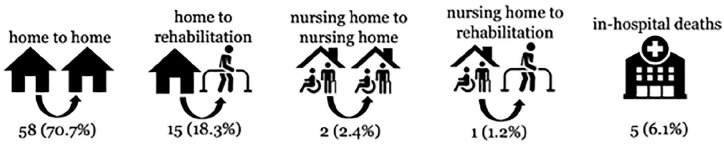
Destinations of discharge with number of patients (%).

Figure 2 shows the living status at admission and the destination of discharge after hospital stay. At discharge, 60 patients (73.2%) were able to return to their preadmission living status, either with or without (additional) home care. Fifteen patients (18.3%) admitted from home, were discharged to a rehabilitation center, which was intended to be temporary. One patient (1.2%) previously living in a nursing home was discharged to a rehabilitation center. It is unknown whether this patient was more care dependent at discharge. No complex EVAR patients were newly admitted to a nursing home postsurgery.

### Functional Performance

Twenty-three out of the 55 complex EVAR patients referred for geriatric assessment gave informed consent for follow-up and were included in the TENT-study for a functional performance analysis (Figure 1).

**Figure 3.**
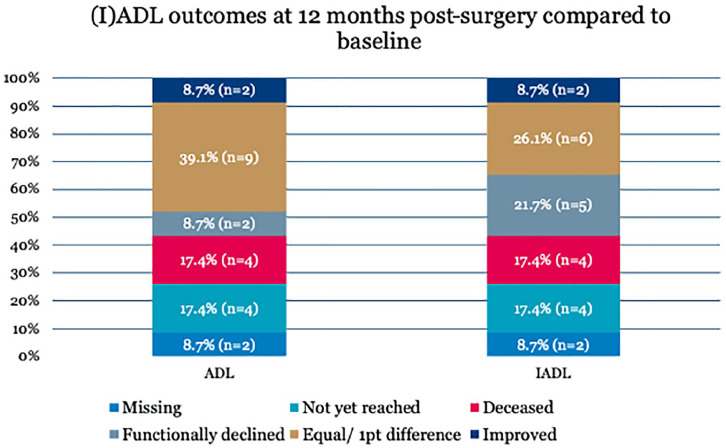
Incidence of functional decline. n (%) in the 23 patients included in the TENT-study at 12 months postsurgery. Functional decline was defined as an increase of at least 2 points on the Katz Activities of Daily Living scale or the Lawton Instrumental Activities of Daily Living scale. ADL, activities of daily living; IADL, denotes instrumental activities of daily living; TENT, Triage of Elderly Needing Treatment.

There was no significant difference between the baseline geriatric scores of patients that did give informed consent for follow-up and patients who did not give consent. Patients who did give informed consent were significantly older compared with patients who did not consent to follow-up (75.6 vs 71.8, p=0.027). Baseline geriatric scores of the 23 patients included in follow-up are depicted in Table 2. The median preoperative ADL score of these 23 patients was 0.0 (IQR=0.0, 1.0). The median preoperative IADL-score was 1.0 (IQR=0.0, 4.0). The MNA showed that 3 patients (13.0%) were at risk for malnutrition. Cognitive impairment, measured by the 6-CIT, was present in 1 out of 23 patients (4.3%). Functional outcomes are depicted in Figure 3. At 12 months, 5 patients presented with functional decline (21.7%), 3 patients with IADL decline only, and 2 patients with IADL as well as ADL decline. ADL decline was mainly caused by the need for assistance in bathing and getting dressed (n=2). IADL decline was mostly caused by needing assistance in shopping (n=3). Mortality at 12 months was 17.4% (n=4). This means that 39.1% of the patients in the care pathway suffered an adverse outcome at 12 months postsurgery.

**Table 2.** Geriatric Scores and Functional Performance of Patients Included in the TENT-study (n=23).

Geriatric Domain	Scores
ADL, median (IQR)	0.0 (0.0, 1.0)
IADL, median (IQR)	1.0 (0.0, 4.0)
Cognitive impaired (6-CIT>7), n (%)	1 (4.3)
At risk for malnutrition (MNA<11), n (%)	3 (13.0)
**Patients with functional decline at 12 mo**, n (%)	
Total	5 (21.7)
IADL decline only	3 (13.0)
IADL and ADL decline	2 (8.7)
Deceased	4 (17.4)

Abbreviations: 6-CIT: 6-item Cognitive Impairment Test; ADL, activities of daily living; IADL, instrumental activities of daily living; IQR, interquartile range (Q1, Q3); MNA, Mini Nutritional Assessment; TENT, Triage of Elderly Needing Treatment.

Medical records of the 5 patients who presented (I)ADL decline were searched for postoperative adverse events that could have caused their functional decline. In 1 patient, a preexistent cognitive disorder worsened during follow-up. No potential function-limiting major complications were registered for this patient. In 2 patients, major complications following complex EVAR were reported: spinal ischemia (n=1) and arterial occlusion of the lower limb (n=1). For 2 patients, no adverse events were registered during follow-up, while no function-limiting major complications of complex EVAR were registered to have occurred.

### Perioperative Outcomes and Complications

Perioperative outcomes are depicted in Table 3. Twenty-four complications with a Clavien-Dindo score of III-IV were registered, which meant that surgical, laparoscopic, or radiological intervention was necessary or a life-threatening complication took place during hospital stay. These 24 complications occurred in 19 patients (23.2%), including 2 out of the 5 patients who presented functional decline. Median length of hospital stay was 7.0 days (IQR=4.5, 12.5). Median length of follow-up was 21.5 months (IQR=4.1, 42.9). Complications that occurred during follow-up are presented in Table 4. Complications were detected in 33 patients (40.2%); the most common complication was aneurysm sac enlargement (n=19, 23.2%). In 11 patients (13.4%), 17 surgical reinterventions were necessary for complications during follow-up, including 1 out of the 5 patients that suffered functional decline. In addition, 12 patients (14.6%) needed endoleak repair.

**Table 3.** Perioperative Outcomes and Surgical Complications.

Variable (unit)	
Surgical complications Clavien-Dindo III-IV	24
Number of patients, n (%)	19 (23.2)
Length of hospital stay in days, median (IQR)	7.0 (4.5, 12.5)
Preadmission living status and destination of discharge, n (%)	
Home to home	58 (70.7)
Home to rehabilitation center	15 (18.3)
Nursing home to nursing home	2 (2.4)
Other/unknown	2 (2.4)
Deceased in hospital	5 (6.1)
Newly admitted to a nursing home	0 (0.0)

Abbreviation: IQR, interquartile range (Q1, Q3).

**Table 4.** Mortality and Complications During Follow-up.

Total deaths during follow-up, n (% of patients)	
Operative deaths (<30 days)	18 (22.0)
In-hospital	6 (7.3)
Within 1 year	5 (6.1)
Causes of death, n (% of deaths)	12 (14.6)
Aneurysm/complex EVAR-related deaths	7 (38.9)
Non-aneurysm related deaths	5 (27.8)
Undefined relation	6 (33.3)
Complications during follow-up, n (%)	
Arterial thrombosis	2 (2.4)
Stent-graft infection	2 (2.4)
Graft migration	1 (1.2)
Aneurysm sac enlargement	19 (23.2)
Stent fracture	3 (3.7)
Aneurysm rupture	0 (0.0)
Other	15 (18.3)
Number of reinterventions, n	29
Patients requiring one or more surgical reinterventions, n (%)	11 (13.4)
Patients requiring endoleak treatment, n (%)	12 (14.6)

Abbreviation: EVAR, endovascular aortic repair.

### Mortality

Mortality numbers are depicted in Table 4. Within 30 days postsurgery, 6 patients (7.3%) died. These patients were treated by FEVAR (n=3), arch-FEVAR (n=2), and BEVAR (n=1). Five deaths were procedure related and occurred in the hospital: intraoperative type A dissection (n=1), pneumonia (n=1), respiratory failure (n=2), and renal failure (n=1). Mortality within 12 months was 14.6% (12 patients). Total mortality during a median follow-up of 21.5 months (IQR= 4.1, 42.9) was 22.0% (18 patients), of which 7 deaths (38.9%) were surgery or aneurysm related.

## Discussion

No patients were discharged to a nursing home and mortality rates were 7.3% and 14.6% at 30 days and 12 months, respectively, for the total patient group. Major adverse events occurred in 23.2% of patients. Patients included in the TENT-study suffered functional decline at 12 months postsurgery in 21.7% (n=5) and 12-month mortality was 17.4%.

The value of these results is difficult to interpret because of the lack of data on functional performance after complex EVAR. We can however compare these results with functional performance after conventional EVAR and OSR. Rectenwald et al^28^ showed that 79.5% of patients who underwent OSR for a thoracoabdominal aneurysm was discharged to home or a rehabilitation facility (91.5% in our study), whereas 20.5% (0% in our study) was newly discharged to a nursing home. Arko et al^29^ reported that 21% of EVAR patients and 25% of OSR patients were not able to shop/travel at the same level prior to surgery at 6-month follow-up. In addition, 4% of OSR patients lost the ability to independently bathe/eat at the same level compared with presurgery.^29^ These results approximate the 21.7% of patients with functional decline at 12 months in our patient cohort. Williamson et al^30^ studied patients who underwent OSR for an infrarenal aneurysm, and reported a decrease in 33% of patients in their functional abilities, including transportation and shopping at a mean follow-up of 34 months. Blomaard et al^31^ showed that 46.2% of patients acutely hospitalized for internal medicine was either deceased of functionally declined 12 months later. For patients considered frail, this percentage was 67.0%.

By including all 82 consecutive patients that underwent complex EVAR, it was attempted to present an unselected “real-world” patient group, commensurate with patients seen in a daily clinical practice. The 30-day mortality rate found in the current study (7.3%) is higher compared with the mortality rate found by Van Calster et al^32^ (4.9%) and lower compared with the 30-day mortality rates mentioned by Oderich et al^6^ (8.2%) and Tran et al^7^ (8.6%). The difference with Van Calster et al^32^ could be explained by different procedures being included. A relatively low percentage of patients was treated for extensive aneurysms using BEVAR (9.2% vs. 25.6% in our study) and no patients were treated using Arch-FEVAR (3.7% in the current study). The use of differing definitions of “complex” EVAR should be kept in mind when interpreting results in the available literature.

This study has several limitations. The number of patients that could be included in the (I)ADL analysis (n=23) is small. However, to our knowledge, this study is the only study that examined functional performance after complex EVAR, using a prospectively maintained database.^33^ Another limitation is formed by potential confounders for functional decline during follow-up, such as adverse events not related to the complex EVAR procedure and aging. Although a fixed (I)ADL decline per year of aging is not established in the available literature, we cannot ignore that with increasing age, functional performance declines.^34,35^ By confining follow-up to 12 months, we attempted to limit the influence of aging as a confounding factor. Possible confounding adverse events were reported descriptively for the functionally declined patients. Surprisingly, some patients improved in function (Figure 3). Given the fact that patients electively treated for aortic aneurysms are usually asymptomatic prior to surgery, this is remarkable. One explanation could be that follow-up (I)ADL scores were self-reported, which could lead to an overestimation bias.^36,37^

It could also be that with treatment of the aneurysm other function-limiting conditions, such as claudication caused by iliac stenosis, improved. In addition, medication use, nutrition, or postoperative home care might have been optimized during hospital stay. This was not further examined in the current study.

When comparing functional performance after conventional EVAR, OSR, and hospitalized older patients in other fields, complex EVAR results are promising. Even more so considering the general frailty of complex EVAR patients, which makes this group often not suitable for OSR. However, for some patients, the prospect of losing independence or not being able to return home after surgery is unacceptable and can be reason to renounce treatment.^10–12^ Therefore, multidisciplinary efforts should be directed toward preventing postoperative functional decline and care dependency.

## Conclusions

The results found in this study give insight in functional performance after complex EVAR. No patients were newly discharged to a nursing home and functional performance results at 12 months are promising. To our knowledge, this is the only study examining functional performance after complex EVAR, by providing data from a prospectively maintained database. Future multidisciplinary research should focus on determining which patients are most prone to deterioration in function, to support treatment decisions and to optimize patient selection, so that efforts can be directed toward preventing postoperative functional decline.

## References

[1] Criado FJ. EVAR at 20: the unfolding of a revolutionary new technique that changed everything. J Endovasc Ther. 2010;17:789–796. 21142491 10.1583/10-3291.1

[2] Parodi JC Palmaz JC Barone HD. Transfemoral intraluminal graft implantation for abdominal aortic aneurysms. Ann Vasc Surg. 1991;5:491–499. 1837729 10.1007/BF02015271

[3] Chaikof EL Dalman RL Eskandari MK , et al. The Society for Vascular Surgery practice guidelines on the care of patients with an abdominal aortic aneurysm. J Vasc Surg. 2018;67:2–77. 29268916 10.1016/j.jvs.2017.10.044

[4] Wanhainen A Verzini F Van Herzeele I , et al. Editor’s Choice—European Society for Vascular Surgery (ESVS) 2019 Clinical Practice Guidelines on the management of abdominal aorto-iliac artery aneurysms. Eur J Vasc Endovasc Surg. 2019;57:8–93. doi:10.1016/j.ejvs.2018.09.020 30528142

[5] Kansagra K Kang J Taon MC , et al. Advanced endografting techniques: snorkels, chimneys, periscopes, fenestrations, and branched endografts. Cardiovasc Diagn Ther. 2018;8:S175–S183. doi:10.21037/cdt.2017.08.17 PMC5949586 29850429

[6] Oderich GS Ribeiro M Reis de Souza L , et al. Endovascular repair of thoracoabdominal aortic aneurysms using fenestrated and branched endografts. J Thorac Cardiovasc Surg. 2017;153:S32–S41. doi:10.1016/j.jtcvs.2016.10.008 27866781

[7] Tran K Lee AM McFarland GE , et al. Complex endovascular aneurysm repair is associated with higher perioperative mortality but not late mortality compared with infrarenal endovascular aneurysm repair among octogenarians. J Vasc Surg. 2019;69:327–333. 29970274 10.1016/j.jvs.2018.04.064

[8] Ultee KHJ Zettervall SL Soden PA , et al. Perioperative outcome of endovascular repair for complex abdominal aortic aneurysms. J Vasc Surg. 2017;65:1567–1575. 28216344 10.1016/j.jvs.2016.10.123 PMC5438879

[9] Yin K Locham SS Schermerhorn ML , et al. Trends of 30-day mortality and morbidities in endovascular repair of intact abdominal aortic aneurysm during the last decade. J Vasc Surg. 2019;69:64–73. 29914839 10.1016/j.jvs.2018.04.032

[10] Solomon MJ Pager CK Keshava A , et al. What do patients want? Patient preferences and surrogate decision making in the treatment of colorectal cancer. Dis Colon Rectum. 2003;46:1351–1357. 14530674 10.1097/01.DCR.0000084432.45536.83

[11] Urbach DR. Measuring quality of life after surgery. Surg Innov. 2005;12:161–165. 16034507 10.1177/155335060501200216

[12] Stegman ME Festen S Brandenbarg D , et al. Using the Outcome Prioritization Tool (OPT) to assess the preferences of older patients in clinical decision-making: a review. Maturitas. 2019;128:49–52. 31561823 10.1016/j.maturitas.2019.07.022

[13] EVAR Trial Participants. Endovascular aneurysm repair and outcome in patients unfit for open repair of abdominal aortic aneurysm (EVAR trial 2): randomised controlled trial. Lancet. 2005;365:2187–2192. 15978926 10.1016/S0140-6736(05)66628-7

[14] Kayssi A DeBord Smith A Roche-Nagle G , et al. Health-related quality-of-life outcomes after open versus endovascular abdominal aortic aneurysm repair. J Vasc Surg. 2015;62:491–498. 26211382 10.1016/j.jvs.2015.05.032

[15] Van Holstein Y van Deudekom FJ Trompet S , et al. Design and rationale of a routine clinical care pathway and prospective cohort study in older patients needing intensive treatment. BMC Geriatr. 2021;21(1):29. 33413165 10.1186/s12877-020-01975-0 PMC7791733

[16] Fletcher GF Ades PA Kligfield P , et al. Exercise standards for testing and training: a scientific statement from the American Heart Association. Circulation. 2013;128:873–934. 23877260 10.1161/CIR.0b013e31829b5b44

[17] Katz S Ford AB Moskowitz RW , et al. Studies of illness in the aged. The index of ADL: a standardized measure of biological and psychosocial function. JAMA. 1963;185:914–919. 14044222 10.1001/jama.1963.03060120024016

[18] Lawton MP Brody EM. Assessment of older people: self-maintaining and instrumental activities of daily living. Gerontologist. 1969;9:179–186. 5349366

[19] Katzman R Fuld P Peck A , et al. Validation of a short Orientation-Memory-Concentration Test of cognitive impairment. Am J Psychiatry. 1983;140:734–739. 6846631 10.1176/ajp.140.6.734

[20] Brooke P Bullock R. Validation of a 6 item cognitive impairment test with a view to primary care usage. Int J Geriatr Psychiatry. 1999;14:936–940. 10556864

[21] Guigoz Y Vellas B Garry PJ. Assessing the nutritional status of the elderly: the Mini Nutritional Assessment as part of the geriatric evaluation. Nutr Rev. 1996;54:59–65. 10.1111/j.1753-4887.1996.tb03793.x 8919685

[22] Ciesla JR Shi L Stoskopf CH , et al. Reliability of Katz’s Activities of Daily Living Scale when used in telephone interviews. Eval Health Prof. 1993;16:190–203. 10125776 10.1177/016327879301600204

[23] Dauphinot V Boublay N Moutet C , et al. Comparison of Instrumental Activities of Daily Living assessment by face-to-face or telephone interviews: a randomized, crossover study. Alzheimers Res Ther. 2020;12:24. 32169093 10.1186/s13195-020-00590-w PMC7068883

[24] Dindo D Demartines N Clavien P. Classification of surgical complications. A new proposal with evaluation in a cohort of 6336 patients and results of a survey. Ann Surg. 2004;240:205–213. 15273542 10.1097/01.sla.0000133083.54934.ae PMC1360123

[25] Lawrence VA Hazuda HP Cornell JE , et al. Functional independence after major abdominal surgery in the elderly. J Am Coll Surg. 2004;199:762–772. 15501119 10.1016/j.jamcollsurg.2004.05.280

[26] Hoogerduijn JG de Rooij SE Grobbee DE , et al. Predicting functional decline in older patients undergoing cardiac surgery. Age Ageing. 2014;43:218–221. 24190876 10.1093/ageing/aft165

[27] Govers AC Buurman BM Jue P , et al. Functional decline of older patients 1 year after cardiothoracic surgery followed by intensive care admission: a prospective longitudinal cohort study. Age Ageing. 2014;43:575–580. 24850542 10.1093/ageing/afu058

[28] Rectenwald JE Huber TS Martin TD , et al. Functional outcome after thoracoabdominal aortic aneurysm repair. J Vasc Surg. 2002;35:640–647. 11932656 10.1067/mva.2002.119238

[29] Arko FR Hill BB Reeves TR , et al. Early and late functional outcome assessments following endovascular and open aneurysm repair. J Endovasc Ther. 2003;10:2–9. 12751922 10.1177/152660280301000103

[30] Williamson WK Nicoloff AD Taylor LM Jr , et al. Functional outcome after open repair of abdominal aortic aneurysm. J Vasc Surg. 2001;33:913–920. 11331828 10.1067/mva.2001.115164

[31] Blomaard LC Lucke JA de Gelder J , et al. The APOP screener and clinical outcomes in older hospitalised internal medicine patients. Neth J Med. 2020;78:25–33. 32043475

[32] Van Calster K Bianchini A Elias F , et al. Risk factors for early and late mortality after fenestrated and branched endovascular repair of complex aneurysms. J Vasc Surg. 2019;69:1342–1355. 30477943 10.1016/j.jvs.2018.08.159

[33] Kärkkäinen JM Sandri GA Tenorio ER , et al. Prospective assessment of health-related quality of life after endovascular repair of pararenal and thoracoabdominal aortic aneurysms using fenestrated-branched endografts. J Vasc Surg. 2019;69:1356–1366. doi:10.1016/j.jvs.2018.07.060 30714570

[34] Brown RT Diaz-Ramirez LG Boscardin WJ , et al. Functional impairment and decline in middle age: a cohort study. Ann Intern Med. 2017;167:761–768. 29132150 10.7326/M17-0496 PMC5716833

[35] Hayase D Mosenteen D Thimmaiah D , et al. Age-related changes in activities of daily living ability. Aust Occup Ther J. 2004;51:192–198.

[36] Schaller A Rudolf K Dejonghe L , et al. Influencing factors on the overestimation of self-reported physical activity: a cross-sectional analysis of low back pain patients and healthy controls. Biomed Res Int. 2016;2016:1497213. doi:10.1155/2016/1497213 27298820 PMC4889825

[37] Janevic MR McLaughlin SJ Connell CM. Overestimation of physical activity among a nationally representative sample of underactive individuals with diabetes. Med Care. 2012;50:441–445. 22193415 10.1097/MLR.0b013e3182422a52 PMC4161147

